# Association of surgical margins with local recurrence in patients undergoing breast-conserving surgery after neoadjuvant chemotherapy

**DOI:** 10.1186/s12885-020-06955-6

**Published:** 2020-05-20

**Authors:** Joseph Lin, Kuo-Juei Lin, Yu-Fen Wang, Ling-Hui Huang, Sam Li-Sheng Chen, Dar-Ren Chen

**Affiliations:** 1 Comprehensive Breast Cancer Center, Changhua Christian Hospital, 135, Nanhsiao Street, Changhua, 500209 Taiwan; 2 Department of Animal Science and Biotechnology, Tunghai University, Taichung, 407302 Taiwan; 3 Department of Surgery, E-Da Hospital, I-Shou University, Kaohsiung, 824410 Taiwan; 4 Cancer Research Center, Department of Research, Changhua Christian Hospital, Changhua, 500209 Taiwan; 5 School of Oral Hygiene, College of Oral Medicine, Taipei Medical University, Taipei, 110301 Taiwan; 6 School of Medicine, Chung Shan Medical University, Taichung, 402367 Taiwan

**Keywords:** Neoadjuvant, Breast-conserving surgery, Surgical margin, Recurrence

## Abstract

**Background:**

The aim of the current study was to report a single-institution experience using breast-conserving surgery after neoadjuvant chemotherapy (NACT), focusing on the association between microscopic resection margin status and locoregional recurrence (LRR).

**Methods:**

Our institutional prospectively maintained database was reviewed to identify patients who were treated with NACT between January 2008 and April 2018.

**Results:**

Among the main partial mastectomy specimens available for analysis (*n* = 161), 28 had margins < 1 mm, 21 had margin width of 1–2 mm and the remaining 112 had margins > 2 mm. LRR occurred in 16 patients (9.9%) and distant metastases were detected in 27 (16.8%) patients. There was no significant difference in the LRR between the > 2 mm margin group with a 60-month cumulative survival of 85.2% compared with 76.2% for the ≤2 mm group (*P =* 0.335) in the Kaplan-Meier analysis. When we stratified patients by margin widths of ≥1 mm or <  1 mm, there was no LRR-free survival benefit observed for the ≥1 mm pathologic excision margin group in the univariate analysis (hazard ratio = 0.443; 95% confidence interval = 0.142–1.383; *P =* 0.161) with a 60-month cumulative LRR-free survival of 84.9% compared with 69.5% for the < 1 mm margin cohort (*P =* 0.150).

**Conclusions:**

In the absence of multiple scattered microscopic tumour foci, a negative margin of no ink on tumour maybe sufficient for stage I–III invasive breast cancer treated with NACT and breast-conserving surgery.

## Background

Despite the lack of overall survival benefits, the use of neoadjuvant chemotherapy (NACT) in early-stage breast cancer nonetheless manifests other advantages; as such, it converts patients into applicants for breast-conserving surgery (BCS) after lowering tumour volumes and reduces the use of axillary lymph node dissection [1, 2]. Moreover, it allows the assessment of therapeutic response to a distinct chemotherapy regimen. The ultimate goals of BCS are complete removal of the breast tumour with adequate margins and simultaneous preservation of the natural shape of the breast [3]. Studies have demonstrated that a “no ink on tumour” lumpectomy margin is adequate for invasive breast cancer treated with BCS followed by whole-breast radiation [4, 5], but those patients display higher locoregional recurrence (LRR) rates than mastectomy patients [6]. Despite the increasing evidence demonstrating the feasibility of BCS after NACT [7], the combined use of NACT and BCS has certainly drawn concerns of high LRR in patients with locally advanced breast cancer as reported by several studies [8–10]. Furthermore, increased pathological complete response (pCR) rates with the use of newer therapeutic agents which was not translated into a higher rate of BCS may have attributed to the distraction in relation to the adequate margin on BCS after NACT [11]. The risk of LRR after BCS could be influenced by factors related to therapeutic strategies, tumour subtypes and surgical margin status. Negative margins reduce the risk of local recurrence, but to date, there is no consensus on what constitutes an adequate negative margin in BCS after NACT. The aim of the current study was to report a single-institution experience using BCS after NACT, focusing on the association between microscopic resection margin status and LRR, as this information can be crucial in improving surgical options after NACT considering the risks and potential benefits in this setting.

## Methods

This study obtained approval from the Changhua Christian Hospital. In this study, patients with breast cancer receiving NACT from January 2008 to April 2018 were enrolled. Initial diagnosis of breast cancer was made through core needle biopsy with ultrasound guidance, through which information on receptor status was obtained using immunohistochemical (IHC) staining. The analysis of estrogen receptor (ER), progesterone receptor (PR), and human epidermal growth factor receptor 2 (HER2) expression by IHC staining was performed on pretherapeutic core needle biopsy specimens. For ER and PR, positivity was defined as expression in 1% of tumour cells. IHC staining with 3+ (moderate to strong complete membrane staining seen in 10% of the tumour cells) or a positive fluorescence in situ hybridization (FISH) test if IHC staining with a score of 2+ (weak to moderate complete membrane in 10% of the tumour cells) was used to determine HER2 positivity. Disease stage was classified based on the seventh edition of the American Joint Committee on Cancer TNM staging system for breast cancer.

Information on Ki-67 expression in pre-therapeutic core needle biopsies was not available until 2018 at our hospital, and histology grade was used as an alternative measurement to determine proliferation activity. Intrinsic subtypes were therefore determined as follows: luminal (ER+ and/or PR+, HER2–, all grades), luminal HER2 (ER+ and/or PR+, HER2+, all grades), HER2-type (ER–, PR– and HER2+) and triple negative (ER–, PR– and HER2–) [12, 13].

Imaging examinations to assess breast and lymph nodes included ultrasonography, mammography and magnetic resonance imaging (MRI); the largest dimension recorded from these examinations was defined as the tumour size. Indications for BCS remained homogenous during the study period: absence of multicentric disease or extensive microcalcification, lack of chest wall or skin involvement and predictable sufficiency of breast volume after BCS. Partial mastectomy specimens were sent to surgical pathologists for microscopic assessment. All patients underwent whole-breast radiation therapy for a total dose of 5000 cGy given in 25–28 fractions with or without a boost to the primary tumour site. Both pre-NACT and post-NACT tumour size were determined by imaging (either MRI or ultrasonography). Our institutional definition of pCR was eradication of invasive cancer and in-situ cancer in the breast and axillary (ypT0, ypN0), which was consistent with the meta-analysis of Cortazar et al. [14]

Histological variants of breast carcinoma were classified into the following subtypes: (1) infiltrating ductal carcinoma (IDC), (2) infiltrating lobular carcinoma (ILC), (3) IDC + ductal carcinoma in situ (DCIS) (DCIS component > 10%), (4) ILC + lobular carcinoma in situ (LCIS) (LCIS component > 10%), (5) IDC + ILC or (6) others (mucinous, medullary, etc.). Primary tumour response to NACT was monitored by ultrasonography after each cycle of chemotherapy, and for tumours that have progressively decreased in size, an ultrasound-guided metallic marker insertion was done for future localisation.

All specimens were oriented with sutures, dye-inked followed by sectioning at 3- to 5-mm intervals, and the smallest distance between the tumour edge and an inked normal tissue margin was measured using an ocular micrometre (to the nearest 1 mm if > 2 mm distance or to the nearest 0.1 mm if < 2 mm). An involved margin was defined as invasive disease at the inked resection margin, whereas uninvolved margins were classified microscopically and reported within a specified distance (> 2 mm, 1–2 mm and <  1 mm) of the resection margin.

The primary outcome of interest was any LRR that was defined as recurrence tumour in the ipsilateral breast parenchyma or metastatic disease in the internal mammary, ipsilateral axillary, infraclavicular or supraclavicular nodes [15]. Secondary outcomes included event-free survival (free of LRR, distant metastasis and death). Time to event was defined as the interval from the definite surgery and the date of the first recurrence.

Clinicopathological characteristics were compared by Mann–Whitney *U* test for medians and chi-square test for proportions. Kaplan–Meier (KM) survival curves were generated to compare the survival outcomes according to the margin status [16], and two-sided log rank test was used to test the significant difference between survival experiences [17]. Statistical analysis was performed using MedCalc statistical software version 18.5 (MedCalc Software bvba, Ostend, Belgium), and a significance level of 5% was used in all analyses.

## Results

**Fig. 1:**
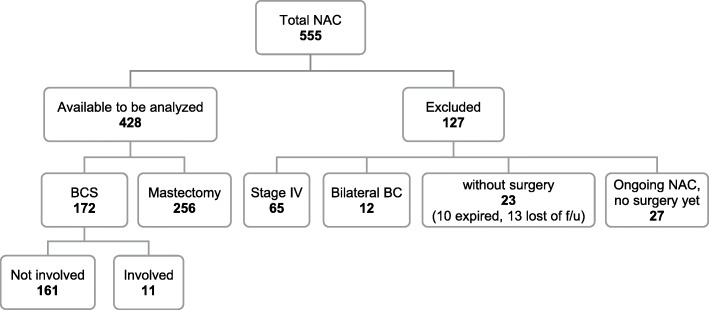
Flow chart of patients treated with NACT followed by surgical treatment

A total of 555 cases were identified, but 127 were excluded because of the following reasons: stage IV breast cancer (*n* = 65), bilateral breast cancer (*n* = 12), lost to follow-up (*n* = 13), expired without surgery (*n* = 10) and on-going NACT (*n* = 27). Of the remaining 428 patients, 172 patients (40.2%) had undergone BCS with radiotherapy and 256 (59.8%) underwent mastectomy (Fig. 1). Of the 172 BCS patients, 11 had involved margin based on pathological examination, and this left us with 161 patients for analysis.

The median age of the studied population was 47.4 years (range 25.4–87.3); 65 (40.4%) patients aged ≥50 years and 96 (50.6%) patients aged < 50 years. NACT comprised of 4–6 courses of anthracycline-based (*n* = 33, 20.5%), taxane-based (*n* = 14, 8.7%), combined anthracycline-taxane-based (*n* = 65, 40.4%) and HER2-targeted agents added regimens (*n* = 46, 28.6%). IDC represented 145 (90.1%) of all patients, which was considered the most common histopathological type in this study. Statistical associations between the three margin groups and tumour characteristics are summarised in Table 1.

**Table 1:** Patient characteristics (*n* = 161)

		Surgical margin			
Characteristics	All (n = 161) (%)	< 1 mm (***n*** = 28) (%)	≥ 1 mm, < 2 mm (***n*** = 21) (%)	≥ 2 mm (***n*** = 112) (%)	***P***
**Age, year**					
Median (range)	47.4 (25.4–87.3)	51.7 (27.6–87.3)	46.4 (25.4–68.5)	46.9 (27.6–74.7)	
Mean ± SD	48.1 ± 11.0	51.7 ± 13.5	46.3 ± 10.1	47.5 ± 10.4	
< 50	96 (59.6)	13 (46.4)	12 (57.1)	71 (63.4)	0.254
≥ 50	65 (40.4)	15 (53.6)	9 (42.9)	41 (36.6)	
**Tumour size**					
T1 (≤ 2 cm)	18 (11.2)	3 (10.7)	1 (4.8)	14 (12.5)	0.782
T2 (> 2 cm, ≤ 5 cm)	132 (82.0)	24 (85.7)	18 (85.7)	90 (80.4)	
T3 (> 5 cm)	11 (6.8)	1 (3.6)	2 (9.5)	8 (7.1)	
**Lymph node status**					
Negative	34 (21.1)	6 (21.4)	5 (23.8)	23 (20.5)	0.944
Positive	127 (78.9)	22 (78.6)	16 (76.2)	89 (79.5)	
**Histological type**					
IDC	145 (90.1)	22 (78.6)	19 (90.5)	104 (92.9)	0.152
IDC + DCIS	12 (7.5)	4 (14.3)	1 (4.8)	7 (6.2)	
Others	4 (2.5)	2 (7.1)	1 (4.8)	1 (0.9)	
**Histological grade**					
in situ	2 (1.3)	1 (3.6)	1 (5.0)	0 (0)	0.173
I	17 (11.3)	4 (14.3)	1 (5.0)	12 (11.8)	
II	77 (51.3)	16 (57.1)	13 (65.0)	48 (47.1)	
III	54 (36.0)	7 (25.0)	5 (25.0)	42 (41.2)	
Missing	11	0	1	10	
**Intrinsic subtype**					
Luminal	53 (32.9)	9 (32.1)	11 (52.4)	33 (29.5)	0.379
Luminal HER2	41 (25.5)	8 (28.6)	3 (14.3)	30 (26.8)	
HER2	21 (13.0)	5 (17.9)	3 (14.3)	13 (11.6)	
TNBC	46 (28.6)	6 (21.4)	4 (19.0)	36 (32.1)	
**Chemotherapy**					
Anthracycline-based	33 (20.5)	5 (17.9)	7 (33.3)	21 (18.8)	0.427
Taxane-based	14 (8.7)	2 (7.1)	2 (9.5)	10 (8.9)	
Combined anthracycline and taxane	65 40.4)	10 (35.7)	7 (33.3)	48 (42.9)	
HER2 targeting agent contained	46 (28.6)	9 (32.1)	5 (23.8)	32 (28.6)	
Others	3 (1.9)	2 (7.1)	0 (0)	1 (0.9)	
**Follow-up, month**					
Median (range)	34.7 (5.3–118.9)	23.5 (5.3–105.5)	39.3 (7.7–105.6)	35.8 (6.9–118.9)	
Mean ± SD	44.9 ± 31.8	36.1 ± 29.5	49.3 ± 34.2	46.2 ± 31.9	

**Fig. 2:**
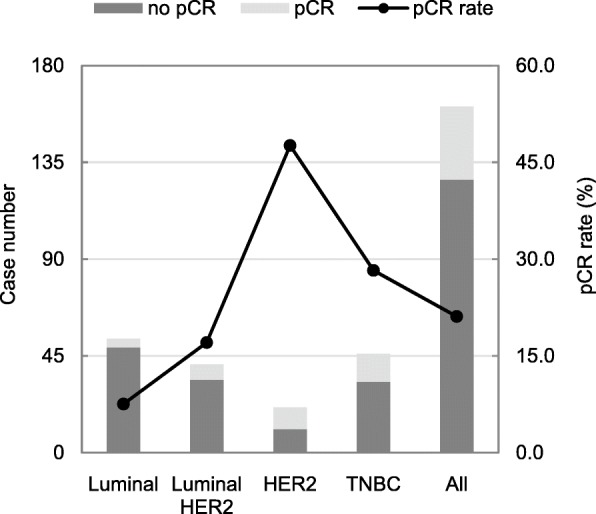
Pathological complete response of NACT patients with BCS by molecular subtypes

Regarding histological grading, 17 cases (11.3%) were grade I, 77 cases (51.3%) were grade II, 54 cases (36%) were grade III and 11 cases did not have grade status. Luminal subtype represented 53 (32.9%) of all patients, triple negative, luminal HER2 and HER2 subtypes represented 46 (28.6%), 41 (25.5%) and 21 (13%) of all BCS patients, respectively. The median follow-up time was 47 months (range 25–87). Thirty-eight patients (22.1%) achieved a pCR; overall pCR was 8.9% (5/56) in luminal subtype patients, 18.2% (8/44) in luminal HER2 subtype patients, 50% (12/24) in HER2 subtype patients and 27.1% (13/48) in triple negative breast cancer (TNBC) patients. Their pCR rates according to molecular subtypes are shown in Fig. 2.

Among the main partial mastectomy specimens available for analysis (*n* = 161), 28 had margins < 1 mm, 21 had margin width of 1–2 mm and the remaining 112 had margins > 2 mm. Involved margins were reported in seven patients, and all of them underwent re-excision to obtain negative margins. Overall, LRR occurred in 16 patients (9.9%) and distant metastases were detected in 27 (16.8%) patients. Of these patients with LRR, an in-breast recurrence developed in 10 patients, five patients had nodal failure and one patient exhibited two sites of LRR simultaneously.

**Fig. 3:**
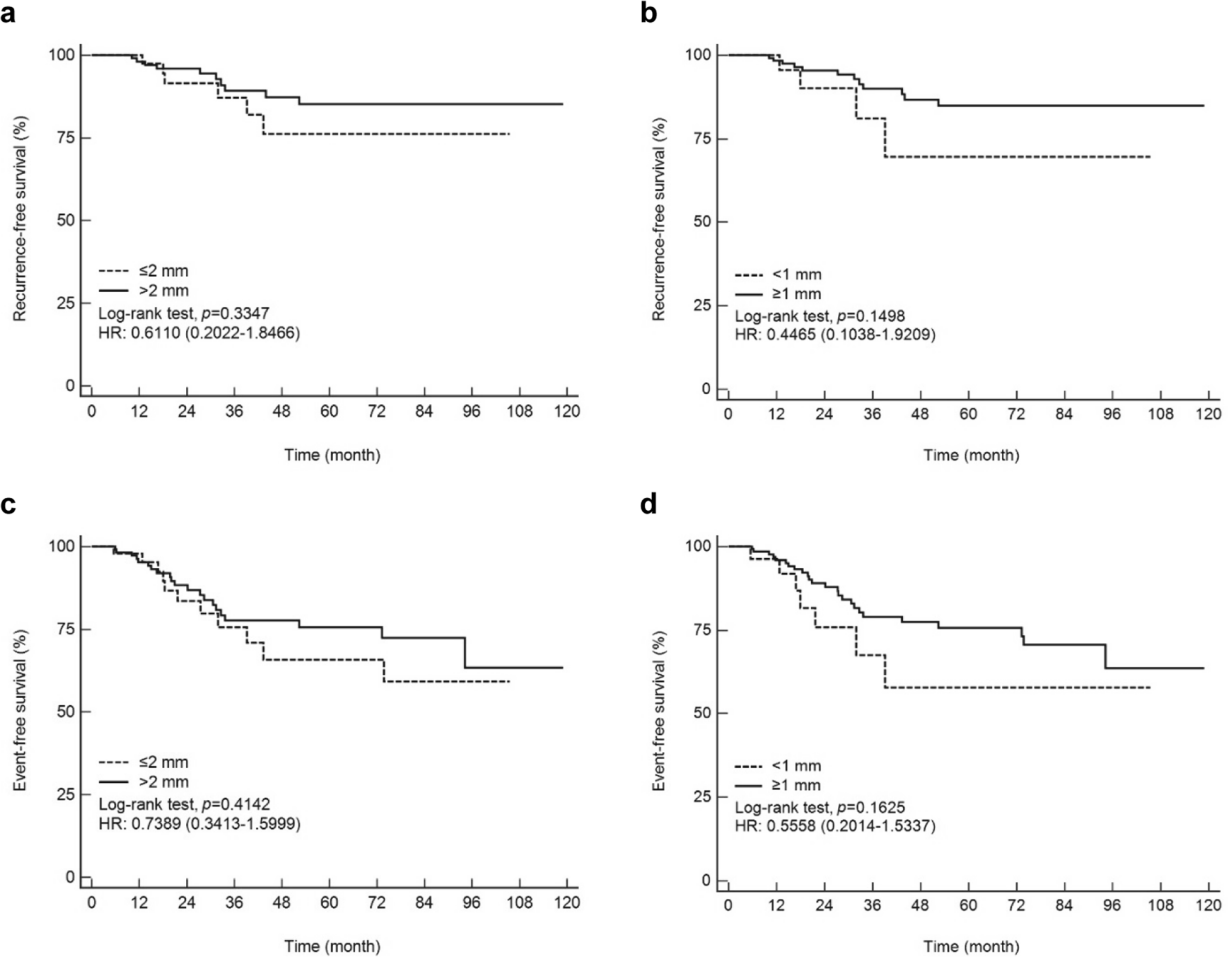
Kaplan–Meier curves. The results demonstrated the relationship between surgical margins and locoregional recurrence-free survival and event-free survival, respectively. **a** and **c**, ≤ 2 mm versus > 2 mm. **b** and **d**, < 1 mm versus ≥1 mm

There were 4 (4/28, 14.3%) LRR events in the < 1 mm margin cohort, 2 (2/21, 9.5%) in the 1–2 mm group and 10 (10/112, 8.9%) in the > 2 mm group. There was no significant difference in the LRR between the > 2 mm margin group with a 60-month cumulative survival of 85.2% compared with 76.2% for the ≤2 mm group (*P =* 0.335; Fig. 3a) in the KM analysis. When we stratified patients by margin widths of ≥1 mm or <  1 mm, there was no LRR-free survival benefit observed for the ≥1 mm pathologic excision margin group in the univariate analysis (hazard ratio = 0.443; 95% confidence interval = 0.142–1.383; *P =* 0.161) (Table 2) with a 60-month cumulative LRR-free survival of 84.9% compared with 69.5% for the < 1 mm margin cohort (*P =* 0.150; Fig. 3b). In the survival analysis for event-free survival, there was no significant difference for margins > 2 mm versus ≤2 mm and no difference for ≥1 mm versus < 1 mm (Fig. 3c-d).

**Table 2:** Univariate logistic regression analysis of LRR-free survival and event-free survival

	LRR-free survival			Event-free survival		
Variables	Hazard ratio	95% confidence interval	***P***	Hazard ratio	95% onfidence interval	***P***
Age, years(≥ 50 vs. < 50)	1.013	0.368–2.789	0.980	0.856	0.413–1.775	0.676
Tumour size, cm(> 2 vs. ≤ 2)	1.270	0.167–9.637	0.817	0.815	0.247–2.686	0.737
Lymph node(positive vs. negative)	2.682	0.609–11.811	0.192	1.972	0.757–5.135	0.164
Histological grade(3 vs. 0–2)	0.851	0.295–2.453	0.766	1.098	0.520–2.316	0.807
ER(positive vs. negative)	0.927	0.334–2.574	0.884	0.884	0.435–1.797	0.734
PR(positive vs. negative)	0.977	0.362–2.633	0.963	0.926	0.456–1.881	0.832
HER2(positive vs. negative)	1.555	0.583–4.146	0.378	1.174	0.583–2.361	0.654
Ki-67 labelling index, %(≥ 14 vs. < 14)	3.253	0.688–15.388	0.137	1.950	0.756–5.034	0.167
Surgical margin, mm(≥ 1 vs. < 1)	0.443	0.142–1.383	0.161	0.554	0.239–1.284	0.169

The logistic regression analysis analyses included age, lymph node status (positive vs. negative), histological grade, receptor status, Ki-67 index, pCR status and surgical margin distance. On the univariate analyses, these variables are independent of LRR-free survival and event-free survival (Table 2). Only lymph node status was found to be a significant predictor of event-free survival (hazard ratio = 3.374; 95% confidence interval = 1.020–11.155; *P =* 0.046) on multivariate analysis.

## Discussion

The introduction of target therapy and advancement of chemotherapeutic treatments have brought an increase in pCR rates, but BCS rates following NACT stay relatively unaffected [11], partly because an increase number of patients may opt for mastectomy treatments due to a lack of consensus on adequate margin in BCS after NACT. These findings may reflect discrepancies in practice among clinicians and guidelines with the consequence of re-excision to gain wider margins.

In the present study, 428 women with untreated operable breast cancer received NACT from January 2008 to April 2018; 40.2% (*n* = 172) of them underwent BCS and the remaining 59.8% (*n* = 256) had mastectomy. The overall BCS rate of 40.2% for patients was lower than that of 49.4% in the surgery first cohort from the previous study [18] despite comparable pCR rates of 22.1% with other studies [19–21]. This may have resulted from the higher LRR after BCS in patients who were treated with NACT [8–10], as one of our senior surgeons who performed more than half of the analysed BCS cases in this study had a BCS rate of 56%. This variation between surgeons in clinical practices further lends credence to the consensus on a safe margin width in this patient population. Furthermore, patients’ decision may have contributed significantly as well. Patients who were eligible for BCS may choose to undergo mastectomy to bypass the subsequent radiation therapy, the risk of higher local recurrence, and the need of intense follow-up.

In our institution, diagnostic ultrasonography was performed by the operating surgeons after each cycle of NACT to evaluate tumour size and therapeutic response. Further, a metallic marker was inserted in tumours that had progressively decreased in size for future localisation. Therefore, we have a lower rate of wire localisation due to an extensive usage of breast ultrasonography, and this low rate does not reflect the simplicity in choosing the optimal resected volume for complete tumour excision while preserving the cosmetic integrity of the breast. Moreover, most patients in this cohort underwent MRI before and after NACT, and this may give additional information in estimation of disease burden during the surgery as other studies suggested [22–24].

Volder et al. [25] reported an involved margin rate of 24.3% in patients who received NACT and BCS, with additional 17.7% of patients with close (≤ 1 mm) margin width identified in a nationwide pathologic study. Differences in therapeutic approaches among hospitals may have contributed to the high-observed margin rate (24.3%) in this population-based study. Others reported a lower rate of re-excision in primary chemotherapy. Christy et al. [26] demonstrated that preoperative chemotherapy resulted in a significantly higher incidence of negative margins (90% vs. 55%; *P* < 0.01) and a lower re-excision rate (6% vs. 37%; *P* < 0.01) compared with primary surgery. Karanlik et al. [27] reported that NACT was more likely to have negative margins (95% vs. 84%; *P =* 0.02) and less likely receive re-excision (4% vs. 8%; *P =* 0.02) as well.

Our study also reported a low re-excision rate, re-excision surgery was given in 4.1% (7/172) of patients, and these seven patients all had an involved margin at the first place. Additional 14.5% (25/172) of patients with margin < 1 mm would have added to this re-excision rate (4.1 + 14.5 = 18.6%) if < 1 mm margin width was considered positive. The lower re-excision rate did not however bring a higher recurrence rate. Moreover, 16 patients (9.9%, 16/161) experienced LRR and 10 of them had breast-only local recurrence (6.2%, 10/161) during the follow-up. Our results were comparable to Mittendorf et al. [28] who reported 5- and 10-years LRR of 7 and 10%, respectively. Our low re-excision rate did not correlate with a higher recurrence rate, and it was further supported by our multivariate analysis that failed to show the association between LRR and margin distance.

A few studies assessed the margin distance and outcomes in patients treated with BCS following NACT, and the results have been inconsistent [10, 19–21] . Chen et al. [19] reported on 340 cases treated at MD Anderson Cancer Centre between 1987 and 2000 and discovered no association between 5-year LRR-free survival and margin distances (> 2 mm vs. ≤ 2 mm). LRR and ipsilateral breast tumour recurrence were correlated with advanced nodal involvement, residual tumour > 2 cm, multifocal residual disease and lymphovascular space invasion. In contrast, the Institute Curie reported that an increased ipsilateral breast tumour recurrence was associated with margins ≤2 mm in addition to clinical tumour > 2 cm, age < 40 and S-phase fraction > 4% [10]. The latest study by Choi et al. [20] on 382 patients showed no association between margin width and local recurrence but rather related to intrinsic subtypes, lack of pCR and positive nodal status. Factors such as age, tumour size, lymph node status, surgical margin, histological grade, Ki-67 index and receptor status were not found to be significant predictors of LRR on univariate analysis in our study. This might have been attributed to our population size due to its insufficient power to detect a difference. However, the low LRR in the present study suggests that even though a statistically significant difference may be achieved by increasing the sample size, this difference may not be translated into a clinically meaningful consequence in the real world.

The rates of pCR were highest in HER2 subtype patients (ER–, PR– and HER2+) with 50% followed by TNBC group with 27.1% in our study. While further analysis on pCR and prognosis stratified by subtypes would be statistically underpowered because of small sample size in our analysis, von Minckwitz in his meta-analysis of 6377 patients treated with NACT and BCS showed different prognosis among pCR patients stratified by subtypes [13]. They reported that pCR was associated with improved disease-free survival in luminal B/HER2 negative, HER2 and TNBC subtypes but not in luminal A or luminal HER2 breast cancer. This may suggest the importance of biologic characteristics of a tumour in achieving local control of breast cancer and the complete resection of the primary tumour may not be essential.

The present study is unique in its analysis of the impact of surgical margins on LRR in Asian patients who underwent NACT followed by BCS which could pose a concern regarding to higher LRR rate than mastectomy [6]. We included patients who were treated recently from 2008 to 2018, during which there has been a constant evolvement and development of breast cancer treatment. While among these earlier mentioned studies [10, 19–21, 24], three analyses were conducted with patients treated with NACT before 2010. It is uncertain if these results still apply to today’s patients treated with current therapy method due to several factors. The advances of systemic therapy and the employment of current radiation technology have yielded better local control and improved results on systematic recurrence. In addition, all patients in the current study underwent preoperative breast MRI examination, and after each cycle of NACT, the surgical surgeon used breast ultrasound examination to assess tumour response. The Evolution of imaging evaluation and modalities may have an effect on reducing margin involvement and local recurrence rates. Lai et al. demonstrated that the combined use of preoperative MRI with conventional breast imaging could lower the surgical involvement rate in patients who received BCS in a case-control comparative analysis [18].

The present analysis included 161 Asian women who underwent NACT followed by BCS and our results suggested the presentation of breast cancer in Asian settings may be different from Western settings. There are nearly 60% of patients in this study who were diagnosed below age of 50 years. Breast cancer at an early age is associated with a greater psychosocial impact because these women may be in the process of finding partners, having children and establishing their careers. Moreover, approximately 90% of patients presented with tumour size ≥2 cm, with the relatively smaller breast volume of Asian patients might make the BCS a less suitable surgical option. Therefore, determining the appropriate oncological margin width is important for Asian women who have small- to moderate-sized breasts to simultaneous preserve the natural shape of the breast which have an effect on the patient’s quality of life subsequently. An earlier survey performed in the United States demonstrated that only 11.2% of surgeons would be satisfied with any negative margin, whereas 47% endorsed > 5 mm [29]. As a result, the attempt to achieve wider margins may regrettably lead to repeat surgery in patients without malignant cells on the inked margin [30]. Moreover, additional surgery (re-excision) is associated with illness, cost, and risk of emotional distress secondary to unsatisfying cosmetic outcomes.

SSO-ASTRO introduced guidelines that suggested “no ink on tumour” as adequate margins for women with invasive breast cancer undergoing BCS, but this analysis did not include patients treated with NACT [4]. The purpose of the current study was to compare margin widths of > 2 mm, 1–2 mm and <  1 mm in NACT patients after BCS and their association with recurrence rate. Our results did not support the idea that ≥1 mm margins would decrease LRR, and it is important because it may reduce additional costs and psychological effect by minimising the need for re-excision [31].

This study had a few limitations. First, this was a retrospective, single-institution study with a comparatively small sample size. Second, the enrolled patients were given different chemotherapy regimens based on the tumour subtype. Additionally, patients with HER2 subtype were underrepresented and it might limit the generalisability of the results. However, this study benefits from its real-world clinical data and a relative standardised strategy to surgical approach and to margin assessment procedure. Further studies with greater sample sizes are necessary to determine the safe surgical margin with NACT and BCS.

## Conclusion

This study has shown no increase in LRR for surgical margins < 1 mm compared with margins ≥1 mm. In the absence of multiple scattered microscopic tumour foci, a negative margin of no ink on tumour maybe sufficient for stage I–III invasive breast cancer treated with NACT and BCS, and it is not necessary for re-excision if surgical width is < 1 mm.

## Data Availability

All datasets used or analysed for this study are available from the corresponding author upon reasonable request.

## Abbreviations

NACT: Neoadjuvant chemotherapy
BCS: Breast-conserving surgery
LRR: Locoregional recurrence
pCR: pathological complete response
PR: Progesterone receptor
MRI: Magnetic resonance imaging
IDC: Infiltrating ductal carcinoma
DCIS: Ductal carcinoma in situ
ILC: Infiltrating lobular carcinoma
LCIS: lobular carcinoma in situ
TNBC: triple negative breast cancer
